# Supplementary material for: Functional divergence of Plexin B structural motifs in distinct steps of Drosophila olfactory circuit assembly
Source: eLife. 2019 Jun 21;8:e48594. doi: 10.7554/eLife.48594 (PMC6597256; doi:10.7554/eLife.48594)
Supplement: Supplementary file 1. [file elife-48594-supp1.docx]

**Supplementary File 1.** Genotypes of flies in each experiment.

| **Figure** | | **Genotype** |
| --- | --- | --- |
| ***Figure 1-figure supplement 1*** | | |
|  | WT: *C155-GAL4/+ (or Y);; UAS-PlexB (WT)/+*  ∆Sema: *C155-GAL4/+ (or Y);; UAS-PlexB (∆Sema)/+*  GAP^mut^: *C155-GAL4/+ (or Y);; UAS-PlexB (GAP^mut^)/+*  ∆Rac1: *C155-GAL4/+ (or Y);; UAS-PlexB (∆Rac1)/+*  Rac1^mut^: *C155-GAL4/+ (or Y);; UAS-PlexB (Rac1^mut^)/+*  ∆Rho1: *C155-GAL4/+ (or Y);; UAS-PlexB (∆Rho1)/+*  Uncleav: *C155-GAL4/+ (or Y);; UAS-PlexB (Uncleav)/+*  Cleav^Sec^: *C155-GAL4/+ (or Y);; UAS-PlexB (Cleav^Sec^)/+*  Cleav^TMCyto^: *C155-GAL4/+ (or Y);; UAS-PlexB (Cleav^TMCyto^)/+* | |
| ***Figure 2*** | | |
| B | Control: *Pebbled-GAL4, UAS-mtdTomato/+ (or Y)*  *plexB^–/–^: Pebbled-GAL4, UAS-mtdTomato/+ (or Y);;; plexB^–^/plexB^–^*  WT: *Pebbled-GAL4, UAS-mtdTomato/+ (or Y);; UAS-PlexB (WT)/+; plexB^–^/plexB^–^* | |
| C | Control: *Pebbled-GAL4, UAS-mtdTomato/+ (or Y)*  *plexB^–/–^: Pebbled-GAL4, UAS-mtdTomato/+ (or Y);;; plexB^–^/plexB^–^*  WT: *Pebbled-GAL4, UAS-mtdTomato/+ (or Y);; UAS-PlexB (WT)/+; plexB^–^/plexB^–^*  ∆Sema: *Pebbled-GAL4, UAS-mtdTomato/+ (or Y);; UAS-PlexB (∆Sema)/+; plexB^–^/plexB^–^* | |
| D | Control: *Pebbled-GAL4, UAS-mtdTomato/+ (or Y)*  *plexB^–/–^: Pebbled-GAL4, UAS-mtdTomato/+ (or Y);;; plexB^–^/plexB^–^*  WT: *Pebbled-GAL4, UAS-mtdTomato/+ (or Y);; UAS-PlexB (WT)/+; plexB^–^/plexB^–^*  GAP^mut^: *Pebbled-GAL4, UAS-mtdTomato/+ (or Y);; UAS-PlexB (GAP^mut^)/+; plexB^–^/plexB^–^* | |
| E | Control: *Pebbled-GAL4, UAS-mtdTomato/+ (or Y)*  *plexB^–/–^: Pebbled-GAL4, UAS-mtdTomato/+ (or Y);;; plexB^–^/plexB^–^*  WT: *Pebbled-GAL4, UAS-mtdTomato/+ (or Y);; UAS-PlexB (WT)/+; plexB^–^/plexB^–^*  ∆Rac1: *Pebbled-GAL4, UAS-mtdTomato/+ (or Y);; UAS-PlexB (∆Rac1)/+; plexB^–^/plexB^–^* | |
| F | Control: *Pebbled-GAL4, UAS-mtdTomato/+ (or Y)*  *plexB^–/–^: Pebbled-GAL4, UAS-mtdTomato/+ (or Y);;; plexB^–^/plexB^–^*  WT: *Pebbled-GAL4, UAS-mtdTomato/+ (or Y);; UAS-PlexB (WT)/+; plexB^–^/plexB^–^*  Rac1^mut^: *Pebbled-GAL4, UAS-mtdTomato/+ (or Y);; UAS-PlexB (Rac1^mut^)/+; plexB^–^/plexB^–^* | |
| G | Control: *Pebbled-GAL4, UAS-mtdTomato/+ (or Y)*  *plexB^–/–^: Pebbled-GAL4, UAS-mtdTomato/+ (or Y);;; plexB^–^/plexB^–^*  WT: *Pebbled-GAL4, UAS-mtdTomato/+ (or Y);; UAS-PlexB (WT)/+; plexB^–^/plexB^–^*  ∆Rho1: *Pebbled-GAL4, UAS-mtdTomato/+ (or Y);; UAS-PlexB (∆Rho1)/+; plexB^–^/plexB^–^* | |
| H | Control: *Pebbled-GAL4, UAS-mtdTomato/+ (or Y)*  *plexB^–/–^: Pebbled-GAL4, UAS-mtdTomato/+ (or Y);;; plexB^–^/plexB^–^*  WT: *Pebbled-GAL4, UAS-mtdTomato/+ (or Y);; UAS-PlexB (WT)/+; plexB^–^/plexB^–^*  Uncleav: *Pebbled-GAL4, UAS-mtdTomato/+ (or Y);; UAS-PlexB (Uncleav)/+; plexB^–^/plexB^–^* | |
| I | Control: *Pebbled-GAL4, UAS-mtdTomato/+ (or Y)*  *plexB^–/–^: Pebbled-GAL4, UAS-mtdTomato/+ (or Y);;; plexB^–^/plexB^–^*  WT: *Pebbled-GAL4, UAS-mtdTomato/+ (or Y);; UAS-PlexB (WT)/+; plexB^–^/plexB^–^*  Cleav^Sec^: *Pebbled-GAL4, UAS-mtdTomato/+ (or Y);; UAS-PlexB (Cleav^Sec^)/+; plexB^–^/plexB^–^* | |
| J | Control: *Pebbled-GAL4, UAS-mtdTomato/+ (or Y)*  *plexB^–/–^: Pebbled-GAL4, UAS-mtdTomato/+ (or Y);;; plexB^–^/plexB^–^*  WT: *Pebbled-GAL4, UAS-mtdTomato/+ (or Y);; UAS-PlexB (WT)/+; plexB^–^/plexB^–^*  Cleav^TMCyto^: *Pebbled-GAL4, UAS-mtdTomato/+ (or Y);; UAS-PlexB (Cleav^TMCyto^)/+; plexB^–^/plexB^–^* | |
| K | Control: *Pebbled-GAL4, UAS-mtdTomato/+ (or Y)*  *plexB^–/–^: Pebbled-GAL4, UAS-mtdTomato/+ (or Y);;; plexB^–^/plexB^–^*  WT: *Pebbled-GAL4, UAS-mtdTomato/+ (or Y);; UAS-PlexB (WT)/+; plexB^–^/plexB^–^*  Cleav^Sec^ + Cleav^TMCyto^: *Pebbled-GAL4, UAS-mtdTomato/+ (or Y);; UAS-PlexB (Cleav^Sec^)/ UAS-PlexB (Cleav^TMCyto^); plexB^–^/plexB^–^* | |
| ***Figure 3*** | | |
| A | Control: *Pebbled-GAL4, UAS-mtdTomato/+ (or Y)*  ORN>PlexB Overexpression: *Pebbled-GAL4, UAS-mtdTomato/+ (or Y);; UAS-PlexB (WT)/+* | |
| B | Control: *Pebbled-GAL4, UAS-mtdTomato/+ (or Y)*  WT: *Pebbled-GAL4, UAS-mtdTomato/+ (or Y);; UAS-PlexB (WT)/+* | |
| C | Control: *Pebbled-GAL4, UAS-mtdTomato/+ (or Y)*  WT: *Pebbled-GAL4, UAS-mtdTomato/+ (or Y);; UAS-PlexB (WT)/+*  ∆Sema: *Pebbled-GAL4, UAS-mtdTomato/+ (or Y);; UAS-PlexB (∆Sema)/+* | |
| D | Control: *Pebbled-GAL4, UAS-mtdTomato/+ (or Y)*  WT: *Pebbled-GAL4, UAS-mtdTomato/+ (or Y);; UAS-PlexB (WT)/+*  GAP^mut^: *Pebbled-GAL4, UAS-mtdTomato/+ (or Y);; UAS-PlexB (GAP^mut^)/+* | |
| E | Control: *Pebbled-GAL4, UAS-mtdTomato/+ (or Y)*  WT: *Pebbled-GAL4, UAS-mtdTomato/+ (or Y);; UAS-PlexB (WT)/+*  ∆Rac1: *Pebbled-GAL4, UAS-mtdTomato/+ (or Y);; UAS-PlexB (∆Rac1)/+* | |
| F | Control: *Pebbled-GAL4, UAS-mtdTomato/+ (or Y)*  WT: *Pebbled-GAL4, UAS-mtdTomato/+ (or Y);; UAS-PlexB (WT)/+*  Rac1^mut^: *Pebbled-GAL4, UAS-mtdTomato/+ (or Y);; UAS-PlexB (Rac1^mut^)/+* | |
| G | Control: *Pebbled-GAL4, UAS-mtdTomato/+ (or Y)*  WT: *Pebbled-GAL4, UAS-mtdTomato/+ (or Y);; UAS-PlexB (WT)/+*  ∆Rho1: *Pebbled-GAL4, UAS-mtdTomato/+ (or Y);; UAS-PlexB (∆Rho1)/+* | |
| H | Control: *Pebbled-GAL4, UAS-mtdTomato/+ (or Y)*  WT: *Pebbled-GAL4, UAS-mtdTomato/+ (or Y);; UAS-PlexB (WT)/+*  Uncleav: *Pebbled-GAL4, UAS-mtdTomato/+ (or Y);; UAS-PlexB (Uncleav)/+* | |
| I | Control: *Pebbled-GAL4, UAS-mtdTomato/+ (or Y)*  WT: *Pebbled-GAL4, UAS-mtdTomato/+ (or Y);; UAS-PlexB (WT)/+*  Cleav^Sec^: *Pebbled-GAL4, UAS-mtdTomato/+ (or Y);; UAS-PlexB (Cleav^Sec^)/+* | |
| J | Control: *Pebbled-GAL4, UAS-mtdTomato/+ (or Y)*  WT: *Pebbled-GAL4, UAS-mtdTomato/+ (or Y);; UAS-PlexB (WT)/+*  Cleav^TMCyto^: *Pebbled-GAL4, UAS-mtdTomato/+ (or Y);; UAS-PlexB (Cleav^TMCyto^)/+* | |
| K | Control: *Pebbled-GAL4, UAS-mtdTomato/+ (or Y)*  WT: *Pebbled-GAL4, UAS-mtdTomato/+ (or Y);; UAS-PlexB (WT)/+*  Cleav^Sec^ + Cleav^TMCyto^: *Pebbled-GAL4, UAS-mtdTomato/+ (or Y);; UAS-PlexB (Cleav^Sec^)/ UAS-PlexB (Cleav^TMCyto^)* | |
| ***Figure 4*** | | |
| A | Control: *Pebbled-GAL4/+ (or Y); Or92a-rCD2/+*  ORN>PlexB Overexpression: *Pebbled-GAL4/+ (or Y); Or92a-rCD2/+; UAS-PlexB (WT)/+* | |
| B | Control: *Pebbled-GAL4/+ (or Y); Or92a-rCD2/+*  WT: *Pebbled-GAL4/+ (or Y); Or92a-rCD2/+; UAS-PlexB (WT)/+* | |
| C | Control: *Pebbled-GAL4/+ (or Y); Or92a-rCD2/+*  WT: *Pebbled-GAL4/+ (or Y); Or92a-rCD2/+; UAS-PlexB (WT)/+*  ∆Sema: *Pebbled-GAL4/+ (or Y); Or92a-rCD2/+; UAS-PlexB (∆Sema)/+* | |
| D | Control: *Pebbled-GAL4/+ (or Y); Or92a-rCD2/+*  WT: *Pebbled-GAL4/+ (or Y); Or92a-rCD2/+; UAS-PlexB (WT)/+*  GAP^mut^: *Pebbled-GAL4/+ (or Y); Or92a-rCD2/+; UAS-PlexB (GAP^mut^)/+* | |
| E | Control: *Pebbled-GAL4/+ (or Y); Or92a-rCD2/+*  WT: *Pebbled-GAL4/+ (or Y); Or92a-rCD2/+; UAS-PlexB (WT)/+*  ∆Rac1: *Pebbled-GAL4/+ (or Y); Or92a-rCD2/+; UAS-PlexB (∆Rac1)/+* | |
| F | Control: *Pebbled-GAL4/+ (or Y); Or92a-rCD2/+*  WT: *Pebbled-GAL4/+ (or Y); Or92a-rCD2/+; UAS-PlexB (WT)/+*  Rac1^mut^: *Pebbled-GAL4/+ (or Y); Or92a-rCD2/+; UAS-PlexB (Rac1^mut^)/+* | |
| G | Control: *Pebbled-GAL4/+ (or Y); Or92a-rCD2/+*  WT: *Pebbled-GAL4/+ (or Y); Or92a-rCD2/+; UAS-PlexB (WT)/+*  ∆Rho1: *Pebbled-GAL4/+ (or Y); Or92a-rCD2/+; UAS-PlexB (∆Rho1)/+* | |
| H | Control: *Pebbled-GAL4/+ (or Y); Or92a-rCD2/+*  WT: *Pebbled-GAL4/+ (or Y); Or92a-rCD2/+; UAS-PlexB (WT)/+*  Uncleav: *Pebbled-GAL4/+ (or Y); Or92a-rCD2/+; UAS-PlexB (Uncleav)/+* | |
| I | Control: *Pebbled-GAL4/+ (or Y); Or92a-rCD2/+*  WT: *Pebbled-GAL4/+ (or Y); Or92a-rCD2/+; UAS-PlexB (WT)/+*  Cleav^Sec^: *Pebbled-GAL4/+ (or Y); Or92a-rCD2/+; UAS-PlexB (Cleav^Sec^)/+* | |
| J | Control: *Pebbled-GAL4/+ (or Y); Or92a-rCD2/+*  WT: *Pebbled-GAL4/+ (or Y); Or92a-rCD2/+; UAS-PlexB (WT)/+*  Cleav^TMCyto^: *Pebbled-GAL4/+ (or Y); Or92a-rCD2/+; UAS-PlexB (Cleav^TMCyto^)/+* | |
| K | Control: *Pebbled-GAL4/+ (or Y); Or92a-rCD2/+*  WT: *Pebbled-GAL4/+ (or Y); Or92a-rCD2/+; UAS-PlexB (WT)/+*  Cleav^Sec^ + Cleav^TMCyto^: *Pebbled-GAL4/+ (or Y); Or92a-rCD2/+; UAS-PlexB (Cleav^Sec^)/ UAS-PlexB (Cleav^TMCyto^)* | |
